# A Review of Graph and Network Complexity from an Algorithmic Information Perspective

**DOI:** 10.3390/e20080551

**Published:** 2018-07-25

**Authors:** Hector Zenil, Narsis A. Kiani, Jesper Tegnér

**Affiliations:** 1 Algorithmic Dynamics Lab, Centre for Molecular Medicine, Karolinska Institute, 171 77 Stockholm, Sweden; 2 Unit of Computational Medicine, Department of Medicine, Karolinska Institute, 171 77 Stockholm, Sweden; 3 Science for Life Laboratory (SciLifeLab), 171 77 Stockholm, Sweden; 4 Algorithmic Nature Group, Laboratoire de Recherche Scientifique (LABORES) for the Natural and Digital Sciences, 75005 Paris, France; 5 Biological and Environmental Sciences and Engineering Division (BESE), King Abdullah University of Science and Technology (KAUST), Thuwal 23955, Saudi Arabia

**Keywords:** algorithmic information theory, complex networks, Kolmogorov-Chaitin complexity, algorithmic randomness, algorithmic probability, biological networks

## Abstract

Information-theoretic-based measures have been useful in quantifying network complexity. Here we briefly survey and contrast (algorithmic) information-theoretic methods which have been used to characterize graphs and networks. We illustrate the strengths and limitations of Shannon’s entropy, lossless compressibility and algorithmic complexity when used to identify aspects and properties of complex networks. We review the fragility of computable measures on the one hand and the invariant properties of algorithmic measures on the other demonstrating how current approaches to algorithmic complexity are misguided and suffer of similar limitations than traditional statistical approaches such as Shannon entropy. Finally, we review some current definitions of algorithmic complexity which are used in analyzing labelled and unlabelled graphs. This analysis opens up several new opportunities to advance beyond traditional measures.

## 1. Introduction

Networks, which are used extensively in science and engineering, are often complex when representing static and dynamic data where edges are relations among objects or events. It is, therefore, of fundamental importance to address the challenge of quantifying this complexity, and their information content to be able to understand, deal with such complexity and eventually steer such objects in educated ways. The ability of a computational model-based analysis of objects to implement a complexity or information-theoretic measure, as shown in [1], is key to understanding the object as well as the capabilities and limitations of the model. For example, popular implementations of lossless compression algorithms used to estimate algorithmic information content such as those based on the Lempel-Ziv (LZ) algorithm can effectively be implemented using Finite State Automata (FSA) [2]. However, this means that they do not hold sufficient computational power to characterize all the features in data [3]. To be able to capture all possible computable (recognizable by computer) properties the full power of compression implied by algorithmic complexity is needed and requires the computational power equivalent to a universal Turing machine not currently present in popular implementations of lossless compression such as LZ. FSAs will therefore only be capable of capturing statistical properties and some basic algorithmic features at the level of regular languages, and so on, other grammars of higher or lower power will cover only a partial subset of all the possible properties that data, such as networks, can display. The use of popular implementations of lossless compression algorithms, which have been widely used to approximate algorithmic complexity is thus, in practice, a very minor improvement over classical Shannon information indexes [1] and can only capture statistical regularities at their respective computational power, i.e., missing relevant algorithmic properties.

In this review, we briefly survey some literature related to information-theoretic approaches to network complexity, but more important, we stress some of the limitations both of current approaches to calculations and applications of classical information theory and algorithmic complexity to graphs and networks. In particular the fragility of entropy in its requirements and its dependencies to associated mass probability distributions and the important limitations of lossless compression used to estimate algorithmic information. Finally, we survey a new avenue and novel directions that attempt to overcome some of these shortcuts.

### 1.1. Notation, Metrics, and Properties of Graphs and Networks

To ensure common ground, in this section, we briefly reprise some common definitions and properties of graphs and complex networks. A vertex labelling *V* of a graph G=(V,E) is a function of the set of vertices *V* to a set of labels different for each vertex. A graph with such a mapping function is called a labelled graph. Otherwise, the graph is said to be unlabelled. |V(G)| and |E(G)| will denote the vertex and edge/link count of *G*.

**Figure 1:**
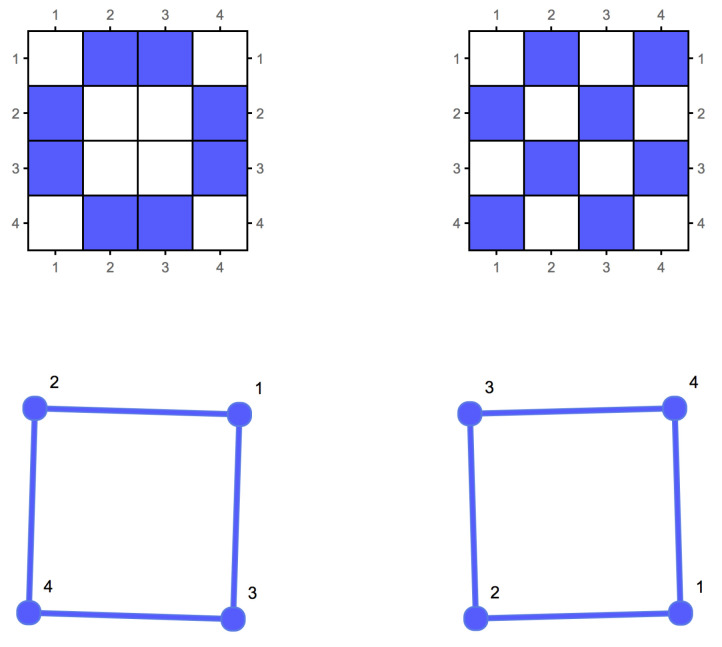
The adjacency matrix is not an invariant description of an unlabelled graph. Two isomorphic graphs can have two different adjacency matrix representations. This translates into the fact that the graphs can be relabelled, thus being isomorphic. However, similar graphs have adjacency matrices with similar algorithmic information content, as proven in [4].

Graphs *G* and *H* are said to be *isomorphic* if there is a bijection between the vertex sets of *G* and *H*, λ:V(G)→V(H) such that any two vertices *u* and v∈G are adjacent in *G* if and only if λ(u) and λ(v) are adjacent in *H*. When *G* and *H* are the same graph, the bijection is referred to as an *automorphism* of *G*. The adjacency matrix of a graph is not an invariant under *graph relabellings*. Figure 1 illustrates two adjacency matrices for isomorphic graphs. A(G) will denote the adjacency matrix of *G*.

The number of links per node constitutes a key characteristic of a graph. When all nodes have the same number of links, the graph is said to be *regular*. The *degree* of a node *v*, denoted by d(v), is the number of links to other nodes.

A *canonical form* of *G* is a labelled graph Canon(G) that is isomorphic to *G*, such that every graph that is isomorphic to *G* has the same canonical form as *G*. An advantage of Canon(G) is that unlike A(G), A(Canon(G)) is a graph invariant of Canon(G) [5].

**Figure 2:**
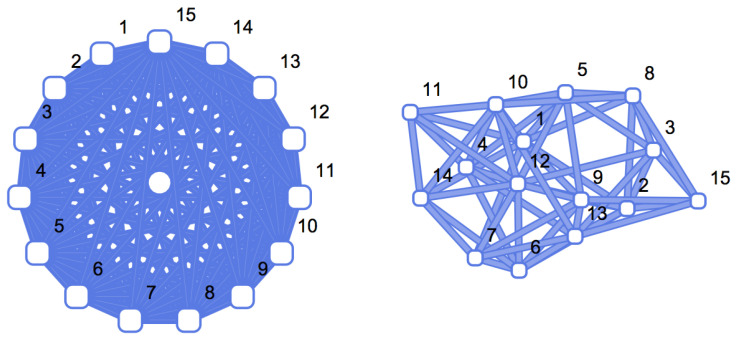
From simple to random graphs. The graphs are ordered based on the estimation of their algorithmic complexity (*K*). $K\left(G\right)∼\log_{2}\left|V\left(G\right)\right|=\log_{2}15∼3.9$ bits when a graph is simple (**left**) and is highly compressible. In contrast, a random graph (**right**) with the same number of nodes and number of links requires more information to be specified, because there is no simple rule connecting the nodes and therefore K(G)∼|E(G)|=15 in bits, i.e., the ends of each edge have to be specified (so a tighter bound would be 2|E(G)|∼30 for an ER graph of edge density ∼0.5.

A popular type of graph that has been studied because of its use as a fundamental random baseline is the Erdős-Rényi [6,7] (ER) graph. Here vertices are randomly and independently connected by links using a fixed prescribed probability (also called *edge density*) (see Figure 2 for a comparison between a regular and a random graph of the same size). The probability of vertices being connected is referred to as the *edge probability*. The main characteristic of random graphs is that all nodes have approximately the same number of links, equal to the average number of links per node. An ER graph G(n,p) is a graph of size *n* constructed by connecting nodes randomly with probability *p* independent of every other edge. Usually edge-independent ER graphs are assumed to be non-recursive (i.e., truly random), but ER graphs can be constructed recursively with, for example, pseudo-random algorithms. Here it is assumed that ER graphs are non-recursive, as theoretical comparisons and bounds hold only in the non-recursive case. For numerical estimations, however, a pseudo-random edge connection algorithm is utilized, in keeping with common practice.

The so-called *small-world* graph describes the phenomenon of many empirical networks where most vertices are separated by a relatively small number of edges. A network is considered to be a *small-world* graph *G* if the average graph distance *D* grows no faster than the log of the number of nodes: D∼log|V(G)|. Many networks are *scale-free*, meaning that their degrees are size independent, in the sense that the empirical degree distribution is independent of the size of the graph up to a logarithmic term. That is, the proportion of vertices with degree *k* is proportional to $\gamma k^{\tau }$ for some τ>1 and constant γ. In other words, many empirical networks display a power-law degree distribution.

### 1.2. Classical Information Theory

Information theory originated in the need to quantify fundamental limits on signal processing, such as communicating, storing and compressing data. Shannon’s concept of information entropy quantifies the average number of bits needed to store or communicate a message. Shannon’s entropy determines that one cannot store (and therefore communicate) a symbol with *n* different symbols in less than log(n) bits. In this sense, Shannon’s entropy determines a lower limit below which no message can be further compressed, not even in principle. A complementary viewpoint on Shannon’s information theory would be to consider it a measure quantifying the *uncertainty* involved in predicting the value of a random variable. For example, specifying the outcome of a fair coin flip (two equally likely outcomes) requires one bit at a time, because the results are independent, each result therefore conveying the maximum entropy. Things begin to get interesting when the coin is not fair. If one considers a coin with heads on both obverse and reverse, then the tossing experiment always results in heads, and the message will always be 1, with absolute certainty.

For an ensemble $X(R,p\left(x_{i}\right))$, where *R* is the set of possible outcomes (the random variable), n=|R| and $p(x_{i})$ is the probability of an outcome in *R*. The Shannon information content or entropy of *X* is then given by

$$H\left(X\right)=-\sum_{i=1}^{n}p\left(x_{i}\right)\log_{2}p\left(x_{i}\right)$$

Thus, calculating H(X) requires the mass distribution probability of ensemble *X*. Here we wish to note that using Shannon’s entropy entails a choice regarding the level of granularity of the analysis. This follows from it being a metric requiring the counting of discrete elements or events. For example, consider the bit string 01010101010101, which clearly has a regular pattern. However, the Shannon entropy of the string at the level of single bits is maximal, as at this level of granularity the string contains the same number of 1s and 0s. Shifting perspective though, the string is clearly regular when two-bit blocks are taken as basic units, instance in which the string has minimal complexity because it contains only 1 symbol (01) from among the 4 possible ones (00, 01, 10, 11). One strategy to mitigate this problem is to take into consideration all possible “granularities” (we call this *Block entropy*), from length 1 to *n*, where *n* is the length of the sequence. This measure is related to what’s also called *predictive information* or *excess entropy* (the differences among the entropies for consecutive block sizes). However, such an approach comes with a computational price tag. To compute the block entropy is prohibitively computationally expensive, as compared to fixing the block size at *n*, as it entails producing all possible overlapping in substrings for all i∈{1,…,n}.

In conclusion, characterizing the complexity or information in a network requires a specification of the level of granularity of the analysis. However, this is exactly what we would like to know or discover for a given complex network. Hence, in this sense we need to assume what we are trying to discover. The block entropy “solution” is to run the analysis across all levels of granularity, which evidently is not a scalable approach. This observation motivates the search for a more unbiased metric. This is indeed a challenging task.

## 2. Classical Information and Entropy of Graphs

One of the major challenges in modern physics is to provide proper and suitable representations of network systems for use in fields ranging from physics [8] to chemistry [9]. A common problem is the description of order parameters with which to characterize the “*complexity of a network*”. Here we note that the issue of order parameters is closely related to the selection of the level of granularity of the analysis, as discussed in the previous section. One common conceptual solution is to perform a selection of the kind of alphabet being used in the analysis of complex networks. Level of granularity or selection of order parameters are examples of ways to meet this challenge. A complementary approach involves using a set of predefined measures to describe a complex network. For example, graph complexity has traditionally been characterized using graph-theoretic measures such as degree distribution, clustering coefficient, edge density, and community or modular structure. In all these cases there are numerous algorithms available which can compute these properties of networks provided one pre-selects the feature of interest that each of these different graph-theoretic indexes can measure.

More recently, networks have also been characterized using classical information theory. One complication is the interdependence of many graph-theoretic properties, which makes measures more sophisticated than single-property measurements [10] difficult to come by. One common approach is to generate graphs that have a certain specific property while being random in all other respects, the rationale being to assess whether or not the property in question is typical among an ensemble of graphs with otherwise seemingly different properties. We have recently advanced methods to improve on this idea underlying the so-called “principle of maximum entropy” or Maxent by way of approximating algorithmic complexity [11] based on the challenge that telling apart pseudo-randomness from algorithmic randomness constitutes and that entropic indexes collapse in the same case yet is fundamental to properly identify.

Indeed, approaches using measures based on Shannon entropy which claim to quantify the information content of a network [12] as an indication of its “typicality” are based on an assumption of associated ensembles provided by the entropy evaluation, the idea of *Maxent* being that the more statistical random the more typical. The claim is that one can construct a “null model” that captures some aspects of a network (e.g., graphs that have the same degree distribution) and see how different the network is from the null model as regards particular features, such as clustering coefficient, graph distance, or other features of interest. The procedure aims at producing an intuition of an ensemble of graphs that are assumed to have been sampled uniformly at random from the set of all graphs with the same property, in order to determine if such a property occurs with high or low probability. If the graph is not significantly different, statistically, from the null model, then the graph is said to be as “simple” as the null model; otherwise, the measure is said to be a lower bound on the “complexity” of the graph as an indication of its random as opposed to causal nature. Yet, to construct a proper null model is far from trivial, since as a rule one does not know what properties are present in the network.

Some applications of entropy are to node and graph degree distributions. For example, a method to estimate upper and lower bounds for extremal node degrees was recently proposed in [13] as a measure of relative entropy calculated from the graph edge probability matrix and largest eigenvalues. Entropy has also been applied to other graph features, such as functions of their adjacency matrices [14], and to distance and Laplacian matrices [15].

A recent example is the computation of the Shannon entropy of adjacency matrices to discover CRISPR candidate regions as a method to transform DNA sequences into graphs [16]. A survey contrasting adjacency matrix-based (walk) entropies and other entropies (e.g., based on degree sequence) is also offered in [14]. The study finds that adjacency-based entropies are more robust vis-à-vis graph size and are correlated with graph algebraic properties, as these are also based on the adjacency matrix (e.g., graph spectrum). However, these walk entropy approaches are designed for static or fixed graphs. For time-varying and evolving graphs other measures have been proposed [17] based on the calculation and change of graph spectral properties.

In estimating the complexity of objects, in particular of graphs, it is common practice to rely on graph- and information-theoretic measures. Here, using integer sequences with properties such as Borel normality, we explain how these measures are not independent of the way in which an object, such as a graph, can be described or observed. From observations that can reconstruct the same graph and are therefore essentially translations of the same description, we will see that when applying a computable measure such as Shannon entropy, not only is it necessary to pre-select a feature of interest where there is one, and to make an arbitrary selection where there is not, but also that more general properties, such as the causal likelihood of a graph as a measure (opposed to randomness) can be largely misrepresented by computable measures such as entropy and entropy rate. Therefore, recursive and non-recursive (uncomputable) graphs and graph constructions based on these integer sequences have been introduced, whose different lossless descriptions have disparate entropy values, thereby enabling the study and exploration of a measure’s range of applications and demonstrating the weaknesses of computable measures of complexity.

One way to describe a network is to use the notion of a node degree sequence of a graph. Clearly, when formulated in this manner, the Shannon entropy can be used to characterize the node degree sequence of a graph, by analogy with strings. This use of entropy was first suggested and introduced by [18]. Similar approaches have been investigated and adopted in characterizing chemical graphs and networks investigated within the computational systems biology community [19]. Yet, a notion of coarse graining is needed, specifically, using the idea of a layered computation of the graph degree distribution such as, for example, a sphere covering. Such an approach provides an hierarchical application of entropy, which can be considered a version of graph traversal entropy rate. Assessing molecular complexity is naturally of considerable interest in chemistry. Here, Shannon entropy has been used to quantify the entropy associated with the degree sequence of the graph reflecting the molecular structure. If the network is unlabelled, then a description using the degree distribution is invariant to relabellings. Hence, the degree distribution is not a lossless representation of a labelled network. This is valid whenever the node labels are not relevant. In contrast, when dealing with time-dependent or temporal networks, the degree distribution cannot be used for analyzing or reconstructing the network from data, as the labels of the nodes, the time-stamps, contain the critical information.

We note that the concept of entropy rate cannot be directly applied to the degree distribution. The reason is that the node degree sequence has no inherent order, because any label numbering will be arbitrary. It therefore follows that Shannon entropy is not invariant vis-à-vis the language description of a network. This is in line with the previous discussion about level of granularity, or, on the same note, order parameters. Please note that a labelled or an unlabelled network has a flat degree distribution and therefore the lowest Shannon entropy for degree sequence and adjacency matrix.

### Fragility of Computable Measures Such as Entropy

Common statistical and computable measures such as Shannon entropy can easily be proven not to be robust and to require arbitrary choices such as coarse graining at multiple levels. Dependent on underlying mass distributions, and mostly quantifying how removed the assumptions of the premises are from real-world applications, Shannon entropy has unfortunately proved to have limited use in dealing with complexity, information content, and ultimately, causation and temporal information. To further our intuition as to why this is the case and assess how to progress beyond it, let us first consider the formal basis of Shannon entropy. In a graph *G* we define the (Shannon) entropy as

$$H\left(A\left(G\right)\right)=-\sum_{i=1}^{n}P\left(A\left(x_{i}\right)\right)\log_{2}P\left(A\left(x_{i}\right)\right)$$

where *G* is the random variable with *n* possible outcomes (all possible adjacency matrices of size |V(G)|). For example, a completely disconnected graph *G* with all adjacency matrix entries equal to zero has entropy H(A(G))=0, since the number of different symbols in the adjacency matrix is 1. However, if the frequency of 1s and 0s differs in A(G), then H(A(G))≠0. In general we will use Block entropy to detect additional graph regularities. The idea is to gloss over the adjacency matrix at different and greater resolution. We note, however, that in calculating the unlabelled Block entropy of a graph one has to consider all possible adjacency matrix representations for all possible labellings. Therefore, the Block entropy of a graph is computed as:

$$H\left(G\right)=\min\{H\left(A\left(g_{L}\right)\right)|G_{L}\in L\left(G\right)\}$$

where L(G) is the group of all possible labellings of *G*.

Other entropy-based measures of network elements are possible. Yet as a rule, they all require that an observer focus on a particular graph element or property of the graph, such as the adjacency matrix, degree sequence, or number of bifurcations. Notably, they do not all converge in entropy, thus illustrating that Shannon entropy is not invariant vis-à-vis different descriptions of the same object. This is in contrast to algorithmic complexity, which has the ability to characterize any general or universal property of a graph or network [20]. Indeed, in [20], it has been recently introduced a graph that is generated recursively by a small computer program of (small) fixed length. Yet when looking at its degree sequence we see that it tends to maximal entropy, and when looking at the adjacency matrix it tends to zero entropy at the limit, thus displaying divergent values for the same object when considering different mass probability distributions—when assuming the uniform distribution to characterize an underlying ensemble comprising all possible adjacency matrices of increasing size, or when assuming all possible degree sequences. In a follow-up paper [21] by an independent group, our techniques were used to find other high entropy graphs generated recursively (and thus actually of low randomness).

The proposed refinement of the so-called principle of maximum entropy—or Maxent—based on algorithmic complexity demonstrates (formally and numerically) how not all ER networks are random [11], and that methods based on algorithmic complexity can to some extent tell apart random from pseudo-random ER networks, calling into question the ability of classical Maxent to compare objects against their most randomized versions.

## 3. Moving Towards Algorithmic Complexity of Graphs

A graph with low entropy has low algorithmic complexity because the statistical regularities found in the graph can be used in a computer program to generate it. However, a graph with high entropy can have high or low algorithmic complexity [20] and the number of high entropy but low algorithmic complexity graphs will diverge [22], this means that entropy overestimates randomness because it can only characterize traditional statistical properties and not algorithmic randomness as it can be directly derived from the works of Kolmogorov [23] and Martin-Löf [24], et al., and as investigated both theoretically and numerically in [1].

Using an entropy-based metric poses also the challenge of selecting an appropriate level of e.g., coarse graining: the problem of knowing which level is the proper level to the challenge of identifying order parameters for a given system to minimize its estimated randomness [22] including the selection of a feature of interest per our discussion of the way in which entropy requires the definition of a variable that belongs to an ensemble with an associated mass probability distribution. It would be optimal if we knew in advance which properties we are after, prior to beginning our search for them. However, how then could we get out of this vicious circle, chasing our own tail in the general case when no feature can be chosen before hand as it is often, if not always, the case in real-world scenarios?

During the last decades we have benefited from pioneering work on the mathematics of algorithmic complexity originating from Kolmogorov [23], Chaitin [25], Solomonoff [26], Levin [27] and Martin-Löf [24], among others. Their framework offers the opportunity, in principle, to analyze complex objects in an unbiased manner from first mathematical principles. That is, from the accepted mathematical definition of randomness by way of algorithmic randomness that is removed from the definition of pseudo-randomness that is so widely used in practice based on classical information theory. Yet, conventionally this approach has been hampered by the notion that since algorithmic randomness is not a computable property, then it is supposed to be of very limited practical value, if any.

### 3.1. Lossless Compression in Network Complexity

It has been traditional to use, misuse and overuse lossless compression algorithms alleging that they provide approximations to algorithmic complexity. While it is true that high compression is a sufficient test for non-randomness, because the length of the compressed file (in bits) together with the fixed length of the lossless compression algorithm (in bits) is an upper bound of algorithmic complexity, implementations popular lossless compression algorithms cannot achieve much better performance than a simple traditional statistics approach very close to what Shannon entropy could achieve by its own [1,3]. It is thus very clear that popular lossless compression algorithms are limited by design, as their aim is to capture statistical regularities (i.e., repetitions) within a sliding window of variable length in order to compile a dictionary where the most frequently repeated long segments are replaced by shorter codes. When such a window length is unbounded, such algorithms are said to be optimal, and even universal [2], as they can approach exact values of entropy rate in the limit. However, they are not optimal or universal in any more fundamental and algorithmic sense. Popular lossless compression algorithms such as LZ implement a look-up table based on a dictionary compiled by assigning the longest repetitions to the shorter codes, something that an algorithmic measure would naturally do, while also identifying regularities beyond statistics. Other lossless compression algorithms are directly based on number of repetitions or Shannon entropy, with most if not all strategies being very similar in essence, being based on counting features, i.e., being what we consider statistical.

Networks have emerged as a unifying language in science and technology. The elements of such graphs range from chemical elements and molecules [19] to interacting computer programs or agents. This language for science has fuelled rapid advances in the development of analytical and computational techniques for describing, deconstructing, and engineering networks. Network theory and algorithms have become key areas, with numerous ramifications into other fields. For example, computational systems analysis in molecular biology has emerged as a conceptual framework within which to understand and reconstruct relations among biological components, a case in point being the construction of transcriptional networks from a gene expression dataset that provides a set of possible hypotheses explaining connections among genes. The regulation of genes (nodes) is captured in the directed edges of the graph. Such knowledge is vital to advancing our understanding of living organisms as systems in specific domains ranging from developmental biology to regenerative medicine. Examples are abundant within biology, medicine, and even health care data.

It is, therefore, essential to provide a fundamental theoretical understanding of networks as objects. Different branches of information theory promise to deliver and develop such a quantitative framework on which we can conceive specific algorithms and applications. Since a natural candidate is Shannon entropy, we will in this review discuss and contrast a Shannon-based approach, lossless compression, and algorithmic information theory, with special reference to networks. One key finding is that a computable measure, using counting and returning an output for every input in finite time, is not invariant vis-à-vis variant descriptions of the object [3].

### 3.2. Alternatives to Lossless Compression

More recently, novel directions have been developed in techniques different to popular lossless compression capable of yielding upper bounds to algorithmic complexity by way of estimating lowe bounds of algorithmic probability [4,28] capable of characterizing algorithmic features by finding the small computer programs that generate a string, a matrix and even a graph. Based on this body of work it has been shown how to avoid the vicious circle referred to above and instead numerically estimate the inherent complexity of objects in general and networks in particular. Thus, complementary to entropy-based measures, new methods to approximate the algorithmic complexity of a graph have been introduced [4,28,29].

Below we provide a brief review of this work illustrating how upper bounds on algorithmic complexity can be estimated with alternatives to popular lossless compression algorithms that are very limited and are closer to Shannon entropy than to algorithmic complexity [1].

### 3.3. Algorithmic Information Theory

Technically, the algorithmic complexity of a string *s* [23,25] is formally defined by

K(s)=min{|p|:U(p)=s}

by invoking a universal Turing machine *U* with an output *s* on halting we obtain the shortest program *p* measured in bits. Here a universal Turing machine *U* is a formal model of a general-purpose computer. Such a machine can be programmed to reproduce any computable object, such as a string. Please note that a network can be considered a set of strings since the graph is defined by the rows or columns of an adjacency matrix. The subscript of *U* can be omitted since, based on the *invariance theorem* [30,31], $K_{U}$ only depends on *U* up to a constant. Technically, ∃γ such that $|K_{U}\left(s\right)-K_{U^{\prime }}\left(s\right)|<\gamma$ where γ is a constant independent of *U* and $U^{\prime }$. Since the theory is asymptotic we have convergence at the limit of long strings (*s*), and the approximations become better the longer. This follows from the invariance theorem. We note that despite our use of different Turing machines *U* and $U^{\prime }$, the results do not depend on the specific Turing machine at the limit. Hence, at the limit (for |s|→∞) the evaluations of the complexity of the string *s* using different Turing machines will coincide.

*K* has the technical inconvenience of being non-computable but, more precisely, it is *semi-computable*, meaning that it can be approximated but is not solvable by algorithmic means. Originally, it has been proven that no effective algorithm exists which takes a string *s* as input and produces the exact integer K(s) as output [23,25]. This is an echo of what is commonly referred to as the undecidability of the halting problem [32] in computer science. This refers to the difficulty of knowing whether or not a given computation will eventually stop. Yet, in practice *K* can be effectively approximated by using, for example, compression algorithms. This fact offers an understanding of algorithmic complexity in terms of uncompressibility. If an object, or a network in our case, is uncompressible, then the network is considered to be an algorithmically random network. If on the other hand, a biological network is compressible, then *K* is small and there exists a shorter description of the network relative to the full network itself. Thus, the degree to which an object can be compressed indicates how removed it is from maximum algorithmic randomness. We can therefore define a compression ratio, also related to the *randomness deficiency*, where the ratio is given by C(G)=Comp(G)/|A(G)|, where Comp(G) is the compressed length in bits of the adjacency matrix *G* of a network using a *lossless* compression algorithm (e.g., Compress). The |A(G)| denotes the size of the adjacency matrix as a block. For example, using the dimensions of the array and multiplying its values such that if the adjacency matrix is 5 × 5, then |A(G)|=25. At this juncture one may ask whether the procedure depends on the nature of the lossless compression algorithm used. If so, then the estimated upper bound would be not be valid in general. The short answer is that it does not depend on the lossless algorithm, since compressibility is a sufficient test for non-randomness. Thus, we can test different lossless algorithms one by one and retain the best compression as an approximation to *K*, as a sequence of lower and lower values of *K*. Naturally, the lossless compression algorithm includes a decompression algorithm that retrieves the exact original object, without any loss of information when decompressed. In summary, the closer C(G) is to 1, the less compressible, whereas the reverse is true if it is close to 0. We note that, interestingly, the fact that a non-computable object such as *K* can be numerically approximated in practice calls to mind historical examples in physics where approximations of complicated integrals have proved to be sufficient in practice.

### 3.4. Algorithmic Probability

The algorithmic probability [26,27,33] of a string *s*, denoted by AP(s), indicates the probability that a valid random program *p* written in bits uniformly distributed produces the string *s* when run on a universal (prefix-free (The group of valid programs forms a prefix-free set (no element is a prefix of any other, a property necessary to keep 0<AP(s)<1.) For details see [30,34].)) Turing machine *U*. Formally,

$$AP\left(s\right)=\sum_{p:U(p)=s}1/2^{\left|p\right|}$$

That is, the sum over all the programs *p* for which a universal Turing machine *U* outputs *s* and halts.

Algorithmic probability and algorithmic complexity *K* are formally (inversely) related by the so-called algorithmic Coding theorem [30,34]:

$$|-\log_{2}AP\left(s\right)-K\left(s\right)|<O\left(1\right)$$

where O(1) is an additive value independent of *s*. Please note that the Coding theorem implies that the algorithmic complexity can be estimated from the frequency of a string.

To illustrate the above let us consider π. Under the assumption of Borel’s absolute normality of π, whose digits appear randomly distributed, and with no knowledge of the deterministic source and nature of π as produced by short mathematical formulae, we ask how an entropy versus an algorithmic metric performs. First, the Shannon entropy rate (thus assuming the uniform distribution along all integer sequences of *N* digits) of the *N* first digits of π, in any base, would suggest maximum randomness at the limit. However, without access to or without making assumptions as regards the probability distribution, approximations to algorithmic probability would assign π high probability, and thus the lowest complexity by the Coding theorem, as has been done in [22,35,36,37,38]. Hence, this illustrates the fundamental difference between the two approaches, as they characterize the object as being strikingly different with regard to their complexity.

Just as with π, it has been proven how certain graphs can be artificially constructed to target any level of Shannon entropy [20,21] and thus any estimation of statistical randomness without changing its algorithmic complexity by means of being recursively generated thus of deterministic nature hence diverging from the entropy values derived from them when the generating source and mass probability distribution is unknown. While this is not a surprise it has been often overlooked in their wide application to all type of objects, in particular, to graphs and networks, as if the measure were meaningful or robust without having to pre-select a feature by choice of associated probability distributions [20].

### 3.5. Approximations to Graph Algorithmic Complexity

It is pertinent to ask how well algorithmic complexity can be estimated, i.e., how well different graphs can be distinguished. For example, having two graphs of the same size, can we distinguish between regular, complex, and random graphs? This was demonstrated in [4] to be feasible for graphs of the same size, and by extension when they grew asymptotically. Here, *K* was calculated using the BDM as a compression algorithm. It assigned low algorithmic complexity to regular graphs, medium complexity to complex networks following Watts-Strogatz or Barabási-Albert algorithms, and higher algorithmic complexity to random networks. This is what we expect theoretically, since random graphs are the most algorithmically complex. Please note that all long binary strings are algorithmically random, and approximately all random unlabelled graphs are algorithmically random [39]. Hence, using algorithmic complexity we can prove that the number of unlabelled graphs is a function of their randomness deficiency, which translates into establishing numerically how distant they are from the maximum value of K(G) and in line with recent proposals to generalize Maxent to algorithmic randomness [11].

As noted above, the *Coding Theorem Method* (CTM) [35,36] provides the means for approximation via the frequency of a string. Now, why is this so? The underlying mathematics originates from the relation specified by algorithmic probability between frequency of production of a string from a random program and its algorithmic complexity. It is also therefore denoted as the algorithmic *Coding theorem*, in contrast to another well known coding theorem in classical information theory). Essentially, the numerical approximation hinges on the fact that the more frequently a string (or object) occurs, the lower its algorithmic complexity. Conversely, strings with a lower frequency have higher algorithmic complexity.

The way to implement a compression algorithm at the level of Turing machines, unlike popular compression algorithms based on Shannon entropy, is to go through all possible compression schemes. This is equivalent to traversing all possible programs that generate a piece of data, which is exactly what the CTM algorithm does.

In [4], numerical evidence was presented supporting the theoretical assumption that the algorithmic complexity of an unlabelled graph should not differ dramatically from any of its labelled versions. This can be understood from the observation that there is a small computer program of fixed size that determines the order of the labelling proportional to the size of the isomorphism group. The size of the isomorphism group makes a difference. Given a large isomorphism group, the labelled networks have more equivalent descriptions, which follow from their symmetries. These can therefore, according to algorithmic probability, be of lower algorithmic complexity.

### 3.6. Reconstructing *K* of Graphs from Local Patterns

For any given network it is of considerable interest to ask whether there are any specific features or patterns in the network. When addressing this challenge we note here the gulf between a pre-selected level of coarse graining versus an “unbiased” algorithmic complexity search for patterns. In what has been referred to as network biology, there is a large body of work originating in the pioneering efforts to discover motifs in networks [40]. Here there is a string analogy to patterns in the DNA string, i.e., motifs determining structures of proteins. In the context of networks, several abundant motifs such as feed-forward and feedback circuits have been discovered. Yet, higher order patterns have been much more difficult to detect due to the exponential increase in the number of possible motifs. Hence a brute force counting strategy is difficult to implement in practice. Yet, these attempts hinge on a search for predefined patterns of a certain size (small number of nodes) which differ relative to the random null model. Alternatively, using the algorithmic complexity framework offers a complementary view of this important problem.

Here we instead determine the algorithmic complexity of a graph. This translates into considering how often the adjacency matrix of a motif is generated by a random Turing machine on a 2-dimensional array, also called a *termite* or *Langton’s ant* [41]. Hence an acounting procedure is performed using Turing machines aiming to approximate the algorithmic complexity of the identified structures. This technique is referred to as the *Block Decomposition Method* (BDM), as introduced in [4] and [22]. The BDM technique requires a partition of the adjacency matrix corresponding to the graph into smaller matrices. With these building blocks in our hands we numerically calculate the corresponding algorithmic probability by running a large set of small 2-dimensional deterministic Turing machines, and then—by applying the algorithmic Coding theorem as discussed above—its algorithmic complexity.

Following such a divide-and-conquer scheme we can then approximate the overall complexity of the original adjacency matrix by the sum of the complexity of its parts. Please note that we have to take into account a logarithmic penalization for repetition, given that *n* repetitions of the same object only add logn to its overall complexity, as one can simply describe a repetition in terms of the multiplicity of the first occurrence. Technically, this translates into the algorithmic complexity of a labelled graph *G* by means of BDM is defined as follows:

(1) $$K_{BDM}\left(G,d\right)=\sum_{\left(r_{u},n_{u}\right)\in A{\left(G\right)}_{d\times d}}\log_{2}\left(n_{u}\right)+K_{m}\left(r_{u}\right)$$

where $K_{m}\left(r_{u}\right)$ is the approximation of the algorithmic complexity of the sub-arrays $r_{u}$ arrived at by using the algorithmic Coding theorem, while $A{\left(G\right)}_{d\times d}$ represents the set with elements $\left(r_{u},n_{u}\right)$, obtained by decomposing the adjacency matrix of *G* into non-overlapping squares, i.e., the block matrix, of size *d* by *d*. In each $\left(r_{u},n_{u}\right)$ pair, $r_{u}$ is one such square and $n_{u}$ its multiplicity (number of occurrences). From now on $K_{BDM}\left(g,d=4\right)$ will be denoted only by K(G), but it should be taken as an approximation to K(G) unless otherwise stated (e.g., when taking the theoretical true K(G) value). Once CTM is calculated, BDM can be implemented as a look-up table, and hence runs efficiently in linear time for non-overlapping fixed size submatrices.

Similar to the notion of Block entropy (c.f. Section 3), the algorithmic complexity of a graph *G* is given by:

$$K^{\prime }\left(G\right)=\min\left\{K\left(A\left(G_{L}\right)\right)|G_{L}\in L\left(G\right)\right\}$$

where L(G) is the group of all possible labellings of *G* whereas $G_{L}$ is a particular labelling. Please note that K(G) provides a choice for graph canonization, since it uses the adjacency matrix of *G* having the lowest algorithmic complexity. Here we combine *G* with the smallest lexicographical representation when the adjacency matrix is concatenated by rows. This is by way of dealing with the fact that *G* does not have to be unique. Next, one may ask how this relates to the findings using a more standard search for motifs, as has been employed in network biology, as discussed above. Specifically, is the BDM approach able to recover known network motifs? To this end we use subarrays of the adjacency matrix in order to ensure that network motifs (over-represented graphs), used in biology and proven to classify superfamilies of networks [40,42], are taken into consideration in the BDM calculation. This demonstrates that BDM alone classifies and identifies the same superfamilies of networks [28] as classical network motifs— as discussed above—were able to identify.

### 3.7. Group-Theoretic Robustness of Algorithmic Graph Complexity

How robust is the measure of algorithmic complexity? Here we review this question by contrasting the computation using unlabelled versus labelled graphs. In short, the metric is robust up to an additive constant. Let us dwell on this issue in some more detail. First, regular graphs have been shown to have low values of *K* whereas random graphs have high estimated values of *K*. This has been shown by actually performing the non-trivial calculation of unlabelled complexity, namely $K^{\prime }$. Furthermore, graphs with a larger set of automorphisms have lower *K* values compared to graphs with a smaller set of automorphisms [4]. Now, an important question is how accurate a labelled estimation of K(G) is with respect to the unlabelled $K^{\prime }\left(G\right)$. This is a valid concern and a useful question to ask since in the general case, the calculation of K(G) is computationally cheap, as compared to $K^{\prime }\left(G\right)$, which carries an exponential overhead. Perhaps surprisingly, the difference $|K\left(G\right)-K^{\prime }\left(G\right)|$ is bounded by a constant. Indeed, as first suggested in [37], there exists an algorithm, denoted by α of fixed length (bit-size) |α| such that all L(G) relabellings of *G* can be computed. This is doable using a brute-force scheme, e.g., by producing all the indicated adjacency matrix rows and their associated permutations of the columns. It therefore follows that $|K\left(G\right)-K\left(G_{L}\right)\left|<|\alpha \right|$ for any relabelled graph $G_{L}$ of *G*. In other words, $K\left(G_{L}\right)=K^{\prime }\left(G\right)+\left|\alpha \right|$, where |α| is independent of *G*. We wish to note here that even if the time complexity of α is commonly believed to not be in the P class, it is not a relevant observation. It is sufficient for the proof to go through that an α exists and is of finite size. We can therefore safely deduce that an estimation of the unlabelled $K^{\prime }\left(G\right)$ by piggy-backing on the estimate of a labelled $K(G_{L})$ is indeed an accurate asymptotic approximation. The brute-force schemata is likely the shortest program description capable of producing all graph relabellings, and therefore the best choice to minimize α.

This result is both relevant and useful in practice. First, we can accurately estimate $K_{L}\left(G\right)$ through K(G) for any lossless representation of *G* up to an additive term. Yet, as noted above, the existence of a finite entity does not readily inform us about the convergence rate of K(G) to $K_{L}\left(G\right)$. Interestingly, numerical estimations demonstrate that the convergence rate is fast. For example, the median of the BDM estimations of all the isomorphic graphs of the graph in Figure 1 is 31.7, with a standard deviation of 0.72. However, when generating a graph, the BDM median is 27.26 and the standard deviation 2.93, clearly indicating a statistical difference. However, more importantly, the probability of a random graph having a large automorphism group count is low, as shown in [4]. These observations are consistent with what we would expect of the algorithmic probability of a random graph—a low frequency of production as a result of running a Turing machine on a 2-dimensional tape. In the current review and in [4] we have also shown that graphs, their formal duals, and their co-spectral versions have similar algorithmic complexity values as estimated using algorithmic probability (BDM). This means that in practice the convergence is not only guaranteed but also sufficiently rapid.

### 3.8. K(G) Is Not a Graph Invariant But Highly Informative

In addition to the issue of robustness discussed in the previous section, here we address what could be referred to as sensitivity. Namely, if two graphs end up with similar K(G), does it follow that they are isomorphic? Let us probe why this is not the case. K(G) can readily be computed via an approximation up to a bounded error that vanishes asymptotically with the increase in the size of the graph. Note, however, that K(G) does not uniquely determine *G*. This is evident from the fact that two non-isomorphic graphs *G* and *H* can have K(G)=K(H). More precisely, the algorithmic Coding theorem provides an estimation of how often this occurs, and it is also related to a simple Pigeonhole argument. Indeed, if *G* or *H* are algorithmically speaking (Kolmogorov-Chaitin) random graphs, then the probability that K(G)=K(H) grows exponentially. If *G* and *H* are complex, then their algorithmic probability $∼1/2^{K}\left(G\right)$ and $∼1/2^{K}\left(H\right)$ respectively are small and are located in the tail of the algorithmic probability distribution, also referred to as the *Universal distribution* or Levin’s *semi-measure*) distribution. This ranges over a very tiny interval of (maximal) algorithmic complexity, hence increasing the chance of collision of the values, i.e., K(G)=K(H).

In [37], we utilized theoretical and experimental estimates of the algorithmic complexity of trivial/simple (denoted here by *S*) and random Erdős-Rényi (ER) graphs. Regular graphs, such as completely disconnected or complete graphs, have algorithmic complexity K(S)=log|V(S)|. ER graphs have maximal complexity, so any other complex network is upper bounded by K(ER) graphs. Finally, note that the algorithmic complexity of a Barabási-Albert (BA) network is low, because it is based on a recursive procedure, while preserving an element of randomness since the generative model comes equipped with an attachment probability.

In [37], theoretical and numerical estimations of algorithmic information content for a range of theoretical and real-world networks is provided. Here, Table 1, offers a larger picture, summarizing the theoretical expectations of the asymptotic behaviour of *K* for different graphs and networks. Notice, that $ER^{\prime }$ represents a graph that satisfies the definition of an ER graph but its edges are not independent because $ER^{\prime }$ is recursively generated by, for example, a pseudo-random number generator (PRNG). This, these discerning capabilities to distinguish randomness from pseudo-randomness and tell apart $ER^{\prime }$ from ER is what the introduction of algorithmic complexity to the study of graphs and networks offers.

To test our numerical approximations to these theoretical estimations we have devised a wide range of experiments, one of the most conclusive with regards to graphs being the one performed on dual and co-spectral graphs. We know that graphs and their duals must have about the same algorithmic complexity because there is a computer program of (small) fixed size that can transform any graph into its dual by simple replacement of edges for nodes and nodes for edges. While for duals it was clear that our methods were numerically sound, the co-spectrality test confirmed the robustness of our measures. For both tests our methods outperformed other approaches, such as compression and Shannon entropy as reported in [22].

## 4. Conclusions

We have surveyed concepts and methods using classical and algorithmic information theory, including properties and limitations of Shannon entropy and lossless compression in the context of graph profiling and a recent direction of research that moves away from classical statistics and the wide application of lossless compression. As surveyed, novel approaches show promising signs and have evolved into alternative tools that are now being applied in the context of temporal dynamic networks with applications to areas such as molecular biology under the name of *algorithmic information dynamics* as introduced in [43] based on the measures surveyed in the second part of this review paper thanks to their robust capabilities in characterizing properties of graphs and networks. These new approaches are important because they are better rooted in the accepted mathematical definition of randomness that make them robust and universal in fundamental ways, as opposed to the more traditional, yet widely exploited, (over)use of statistical randomness in either its Shannon entropy or popular lossless compression forms. These new approaches are thus better equipped to deal with more difficult challenges such as that of causation removed from traditional correlation.

## Figures and Tables

**Table 1:** Theoretical calculations of *K* for different network topologies for 0≤p≤1. Clearly, minimum values are for fully connected, fully disconnected and recursive graphs while maximum *K* is reached for edge-independent ER graphs with edge density p=0.5 and fixed number of nodes for which K(ER)∼(|V(ER)|2)/2. For WS graphs, *p* is the rewiring probability.

Type of Graph/Network	Asymptotic Expected Behaviour
Empty/Complete (E)	K(E)∼log|V(E)|
Regular recursive (R) (e.g., cycles, stars)	K(R)∼log|V(R)|
Barabási-Albert (BA)	K(BA)∼|V(BA)|+c
Watts-Strogatz (WS)	$\lim_{p\to 0}K\left(WS\right)∼K\left(R\right)$
	$\lim_{p\to 1}K\left(WS\right)∼K\left(ER\right)$ or $K(ER^{\prime })$
Algorithmic random Erdős-Rényi (ER)	$K\left(ER\right)∼\frac{n(n-1)}{16p|p-1|}$
Pseudo-random Erdős-Rényi $\left(ER^{\prime }\right)$	$K\left(ER^{\prime }\right)∼K\left(S\right)$
